# Design and physicochemical characterisation of novel dissolving polymeric microneedle arrays for transdermal delivery of high dose, low molecular weight drugs

**DOI:** 10.1016/j.jconrel.2014.02.007

**Published:** 2014-04-28

**Authors:** Maelíosa T.C. McCrudden, Ahlam Zaid Alkilani, Cian M. McCrudden, Emma McAlister, Helen O. McCarthy, A. David Woolfson, Ryan F. Donnelly

**Affiliations:** a Queen's University, Belfast School of Pharmacy, 97 Lisburn Road, Belfast BT9 7BL, UK; b School of Pharmacy, Zarqa University, Zarqa 132222, Jordan

**Keywords:** Microneedles, Transdermal, Ibuprofen, Biocompatibility

## Abstract

We describe formulation and evaluation of novel dissolving polymeric microneedle (MN) arrays for the facilitated delivery of low molecular weight, high dose drugs. Ibuprofen sodium was used as the model here and was successfully formulated at approximately 50% w/w in the dry state using the copolymer poly(methylvinylether/maleic acid). These MNs were robust and effectively penetrated skin *in vitro*, dissolving rapidly to deliver the incorporated drug. The delivery of 1.5 mg ibuprofen sodium, the theoretical mass of ibuprofen sodium contained within the dry MN alone, was vastly exceeded, indicating extensive delivery of the drug loaded into the baseplates. Indeed in *in vitro* transdermal delivery studies, approximately 33 mg (90%) of the drug initially loaded into the arrays was delivered over 24 h. Iontophoresis produced no meaningful increase in delivery. Biocompatibility studies and *in vivo* rat skin tolerance experiments raised no concerns. The blood plasma ibuprofen sodium concentrations achieved in rats (263 μg ml^− 1^ at the 24 h time point) were approximately 20 times greater than the human therapeutic plasma level. By simplistic extrapolation of average weights from rats to humans, a MN patch design of no greater than 10 cm^2^ could cautiously be estimated to deliver therapeutically-relevant concentrations of ibuprofen sodium in humans. This work, therefore, represents a significant progression in exploitation of MN for successful transdermal delivery of a much wider range of drugs.

## Graphical abstract

**Figure:**
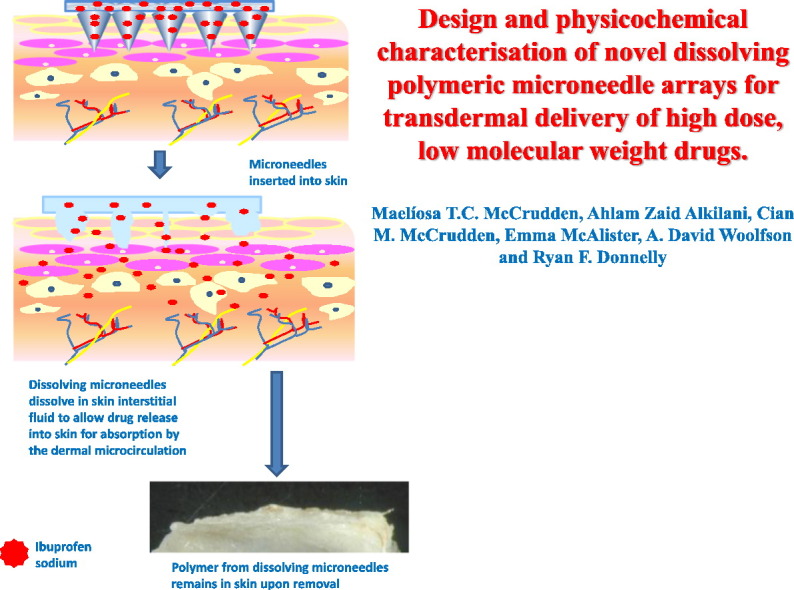

## 1 Introduction

Microneedle (MN) arrays are micron scale, minimally-invasive devices that painlessly by-pass the skin's *stratum corneum* (*SC*), which is the principal barrier to topically-applied drugs. MN arrays have been extensively investigated in recent years as a means to enhance transdermal drug and vaccine delivery. The current trend in MN-based research has involved recognition of the dubious biocompatibility of silicon and the potential for inappropriate reuse of silicon or metal microneedles, which remain fully intact after removal from a patient's skin. Consequently, much recent effort has focussed on MN arrays prepared from drug-loaded gels of FDA-approved biocompatible polymers. Such systems typically dissolve in skin interstitial fluid to release their drug payload.

Dissolving MN arrays have been shown to enhance transdermal and intradermal delivery of numerous substances, including insulin [1,2], 5-aminolevulinic acid [3], sulforhodamine B [4], low molecular weight heparin [5], ovalbumin [6,7], adenovirus vector [7] and a variety of vaccine antigens [8,9]. Synergistic effects of dissolving MN arrays used in combination with other enhancing strategies have been reported recently by Garland et al. [10], where the use of drug-loaded dissolving poly(methyl-vinyl-ether-co-maleic-acid) MN arrays was coupled with iontophoresis.

**Fig. 1:**
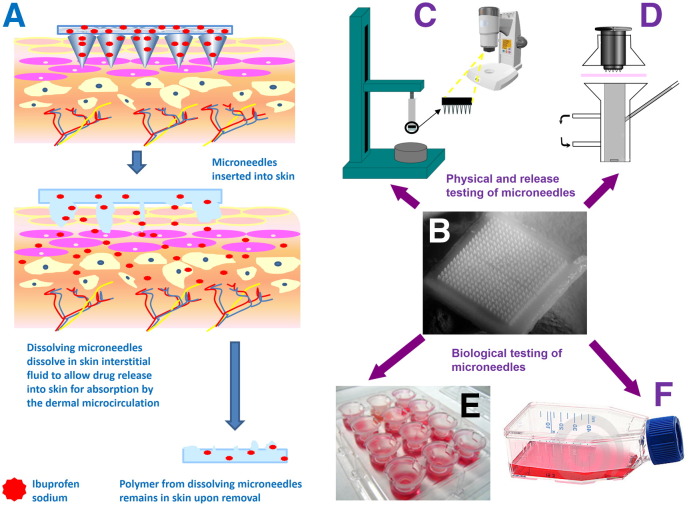
Schematic illustration of the mechanism of drug delivery from dissolving microneedle arrays with containing ibuprofen sodium (A). Digital image of the optimised formulation for dissolving microneedles containing ibuprofen sodium (B). Texture Analyser/light microscopy set-up for investigation of physical properties of microneedles (C) and Franz cell set-up for *in vitro* transdermal drug release studies (D). Indication of biological testing of microneedles in 3D (E) and 2D (F) cell culture models.

A schematic depiction of the means by which dissolving MN arrays deliver their payload is presented in Fig. 1(A). The compounds delivered to date by dissolving MNs have typically been of high potency, meaning only a low dose is required to achieve a therapeutic affect (*e.g.* insulin) [11] or elicit the required immune response [8,9]. Accordingly, dissolving MN arrays have proven to be an extremely successful delivery strategy, even though high molecular weight biomolecules are only normally delivered from the dissolving MNs themselves and not the baseplate upon which they are formed [11]. Clearly, the majority of marketed drug substances are not low dose high potency biomolecules. Indeed, many drugs require oral doses of several hundred milligrams per day in order to achieve therapeutic plasma concentrations in humans. Until now, such high doses could not be delivered transdermally from a patch of reasonable size, even for molecules whose physicochemical properties are ideal for passive diffusion across the skin's *stratum corneum* barrier. Therefore, transdermal delivery has traditionally been limited to fairly lipophilic low molecular weight, high potency drug substances. Since most drug substances do not possess these properties, the transdermal delivery market has not expanded beyond around 20 drugs [12–14]. In the present study, we aimed to overcome the current limitations of both conventional transdermal delivery and dissolving MN strategies to deliver, for the first time, therapeutically-relevant doses of a model low molecular weight, high dose drug molecule.

## 2 Materials and methods

### 2.1 Chemicals

Polyethylene glycol (PEG, MW 10,000 Da), ibuprofen sodium, poly(vinyl alcohol) (PVA, MW 31,000–50,000 g/mol), polyvinylpyrrolidone (PVP, MW 40,000 g/mol), alginic acid sodium salt and the 3-(4,5-dimethyl-2-thiazolyl)-2,5-diphenyl-2H-tetrazolium bromide (MTT) cell viability reagent were purchased from Sigma Aldrich, Dorset, UK. Eudragit® S (MW 125,000 g/mol) and Eudragit® L (MW 125,000 g/mol) were obtained from Rohm GmbH & Co.KG, Pharma Polymers, Darmstadt, Germany. Poly(lactic acid) (PLA) was purchased from Futerro, Escanaffles, Belgium. Isocratic HPLC grade methanol and acetonitrile were purchased from VWR International, East Grinstead, UK. L-132 lung epithelial cells were purchased from the American Type Culture Collection (ATCC) and EpiSkin™ was purchased from Skin Ethic Laboratories, Lyon, France. The human IL-1α ELISA kit and Bradford assay kit were purchased from Pierce, Rockford, IL, USA. Gantrez® AN-139, a co-polymer of methyl vinyl ether and maleic anhydride (PMVE/MAH, MW 1,080,000 Da) and Gantrez® MS-955, a mixed sodium and calcium salt of methyl vinyl ether and maleic anhydride copolymer (PVM/MA, MW 1,000,000 Da) were gifts from Ashland, Kidderminster, UK. All other chemicals used were of analytical reagent grade.

### 2.2 Microneedle array fabrication

Laser-engineered silicone micromould templates were used in micromoulding of MN arrays and were microfabricated using a previously-reported approach [15]. The arrays were composed of 361 (19 × 19) needles perpendicular to the base, of conical shape and 600 μm in height, with base width of 300 μm and interspacing of 50 μm. The array area was approximately 0.49 cm^2^. In order to test the compatibility and suitability of a number of different polymers as potential matrices in the formation of polymeric MN arrays with high loadings of incorporated ibuprofen sodium, various aqueous gel formulations were prepared, as summarised in Table 1. Approximately 300 mg of the relevant polymer gel/drug preparation was poured into the silicone moulds and these were centrifuged for 15 min at 550 ×*g*. Following centrifugation, the MN arrays were dried in the moulds at room temperature for 48 h. The MN arrays were then carefully removed from the moulds and assessed visually for mechanical strength and formulation homogeneity.

### 2.3 Fabrication of PMVE/MA microneedle arrays incorporating ibuprofen sodium

After numerous iterations, MN arrays prepared using the free acid co-polymer poly(methylvinyl ether/maleic acid) (PMVE/MA), produced by aqueous hydrolysis of the PMVE/MAH supplied as described previously [11,15,16], were found to have superior properties to other compositions (Fig. 1B). To prepare such arrays, relevant masses of ibuprofen sodium and a 30% w/w PMVE/MA gel, the pH of which had been altered to 7.0 using sodium hydroxide (NaOH) pellets, were added together so as to generate a formulation of polymer gel:drug in the ratio 70%:30%. This formulation was then poured into the silicone micromoulds, centrifuged for 15 min at 550 ×*g* and again allowed to dry under ambient conditions for 48 h.

### 2.4 Rheological characterisation of PMVE/MA gels containing ibuprofen sodium

In order to consider the processability of gels with such high drug loadings, continuous flow rheological assessment of the gels was performed using a TA Instruments AR 1500 Rheometer (TA Instruments, Elstree, Herts, UK) fitted with a 40 mm diameter steel parallel plate. Flow rheology was conducted at 25 °C in continuous ramp mode with the shear rate increased from 0 to 50 1/s. Viscosity was determined by application of the Power law.

### 2.5 Determination of water content of PMVE/MA microneedles incorporating ibuprofen sodium

The percentage water content of the ibuprofen sodium-loaded PMVE/MA MN arrays was determined with a Q500 Thermo Gravimetric Analyser (TA Instruments, Elstree, Herts, UK). Samples of 5.0–10.0 mg were heated from ambient temperature to 600 °C at a heating rate of 10 °C min^− 1^. Nitrogen flow rates of 40 ml min^− 1^ (balance purge gas) and 60 ml min^− 1^ (sample purge gas) were maintained for all samples. The data from thermogravimetric analysis experiments was analysed with TA Instruments Universal Analysis 2000 software, version 4.4A (TA Instruments, Elstree, Herts, UK).

### 2.6 Mechanical testing of microneedle arrays

MN arrays were subjected to mechanical tests for compression and skin insertion. The mechanical properties were evaluated using a TA-XT2 Texture Analyser (Stable Microsystems, Haslemere, UK) in compression mode, as described previously [15] (Fig. 1C). MN arrays were visualised before and after application of the compression load using a light microscope (GXMGE-5 digital microscope, Laboratory Analysis Ltd., Devon, UK).

To investigate insertion properties, neonatal porcine skin, previously determined to be a good model for human skin in terms of hair sparseness and physical properties [17,18] was obtained from stillborn piglets and immediately (< 24.0 h after birth) excised and trimmed to a thickness of 350 μm using an electric dermatome (Integra Life Sciences™, Padgett Instruments, NJ, USA). Skin was then stored at − 20 °C until required. Before performing penetration studies, the skin was carefully shaved using a disposable razor and stained with methylene blue aqueous solution (1% w/v). The solution was gently wiped off, with dry tissue paper and then with saline and alcohol swabs. The skin was placed, dermis side down, on a 500 μm-thick sheet of dental wax (Anutex^®^, Kemdent Works, Swindon, UK) topped with Parafilm^®^ (Alpha Laboratories, Hampshire, UK) and this assembly was then secured on a wooden block for support. Using double-sided adhesive tape, MN arrays were carefully attached to the moveable cylindrical probe. The probe was lowered onto the skin at a speed of 0.5 mm s^− 1^ until the required force was exerted. Forces were held for 30 s with known forces of 0.4 and 0.5 N per needle. Once the target force was reached, the probe was moved upwards at a speed of 0.5 mm s^− 1^. After removal of MN arrays, the skin was viewed under the digital microscope. The surface of the stained skin was then photographed using the digital camera and the number of microconduits was determined visually.

### 2.7 Determination of ibuprofen sodium recovery from MN arrays

In order to determine the percentage recovery of ibuprofen sodium from dissolving MN arrays, the arrays were dissolved in 100 ml PBS (pH 7.4) in a volumetric flask and two 10-fold dilutions were subsequently carried out in PBS. The drug content of each MN array was determined by HPLC as described below and the percentage recovery of ibuprofen sodium was then determined.

### 2.8 Microneedle in-skin dissolution kinetics

The dissolution rate of the ibuprofen sodium-loaded PMVE/MA MN arrays was investigated in dermatomed neonatal porcine skin. The skin samples were carefully shaved using a disposable razor and then equilibrated in PBS (pH 7.4) for 15 min. A circular specimen of the skin was then dried and carefully secured to the donor compartment of a modified Franz diffusion cell (FDC-400 flat flange, 15 mm orifice diameter, mounted on an FDCD diffusion drive console providing synchronous stirring at 600 rpm and thermostated at 37 ± 1 °C, Crown Glass Co. Inc., Sommerville, NJ, USA) using cynoacrylate glue (Loctite Ltd, Dublin, Ireland) with the *stratum corneum* accessible *via* the top of the donor compartment. This was then placed on a piece of dental wax to support the skin and MN arrays were inserted into the centre of the skin section using a custom-made applicator device (11.0 N force) [15]. To ensure the MN arrays remained within the skin, a circular steel weight (diameter 11.0 mm, 5.0 g mass) was placed on top of the MN arrays. With the arrays in place, donor compartments were mounted onto the receptor compartments of the Franz cells. At the indicated time points, the arrays were removed from the skin, flash frozen in liquid nitrogen, and stored at − 20 °C until viewing. MN arrays were viewed using a Leica MZ6 dissection microscope (Leica Microsystems (UK) Ltd., Milton Keynes, UK) fitted with a Nikon Coolpix 950 digital camera (Nikon UK Limited, Surrey, UK).

### 2.9 *In vitro* drug delivery studies

Diffusion of ibuprofen sodium released from dissolving PMVE/MA MN arrays across dermatomed (350 μm) neonatal porcine skin was investigated *in vitro* using the modified Franz diffusion cells, as described previously [11] (Fig. 1D). The MN array was placed on top of the skin and again inserted using the custom-made applicator (11 N). A piece of occlusive dressing (Scotchpak™ 9732, 3 M, Carrickmines, Ireland, coated with a 1.0 mm layer of DuroTak™ 34-416A, National Starch & Chemical Company, Bridgewater, NJ, USA), identical to that used in the *in vivo* studies described below, was used to secure the MN arrays in place. The cylindrical 5.0 g stainless steel weight was again placed on top of the occluded array and the donor compartment of the apparatus was clamped onto the receiver compartment. The donor compartment and sampling arm were sealed using Parafilm®. The receiver compartment contained PBS (pH 7.4) degassed prior to use and thermostated to 37 ± 1 °C. Syringes (1.0 ml) with 8.0 cm needles were used to remove 200 μl of the Franz cell contents at appropriate time points and 200 μl of pre-warmed PBS was subsequently added to replace this. Samples were centrifuged for 5 min at 14,000 ×*g* using an Eppendorf Minispin centrifuge (Eppendorf UK Limited, Stevenage, UK) and appropriately diluted in PBS prior to HPLC analysis.

In *in vitro* studies exploring the combinatorial approach of iontophoresis and dissolving MN arrays, a silver wire (acting as the anode) was placed into the receiver medium *via* the side arm of the Franz cell and a silver–silver chloride electrode (acting as the cathode) was placed on top of the dissolving MN array. A commercially available power supply (Phoresor II, Iomed, Salt Lake City, FL, USA) was used to deliver a current of 0.5 mA cm^− 2^ over a 6 h period. Sampling and analysis were performed as described above.

### 2.10 Biocompatibility studies

Since both polymer and drug are deposited in skin by dissolving MN, their influence on cellular viability and irritancy are of great interest. Initial studies here were *in vitro*, with both 2D and 3D cell culture models employed. Human L-132 lung cells, engaged as an epithelial cell model in this study, were routinely maintained in Minimum Essential Medium (MEM, Invitrogen, Paisley, UK) supplemented with 10% foetal bovine serum (FBS). Cells were cultured in tissue culture plasticware (Thermo Scientific, Waltham, MA, USA) and housed in a humidified incubator at 37 °C, with a CO_2_ content of 5% (Fig. 1F).

Sodium dodecyl sulphate (SDS) was used as a positive control, as it has long been recognised as a human skin irritant [20] and has been demonstrated to induce the production and release of the irritancy biomarker, interleukin-1α [IL-1α] [21,24]. All test compounds (ibuprofen sodium, PMVE/MA, ibuprofen sodium-loaded PMVE/MA MN arrays) were dissolved in cell culture medium and were filter-sterilised using a 0.2 μm filter prior to their addition to the cells. The negative control was cell culture medium alone. During cell viability and IL-1α expression analysis experiments, L-132 cells, at a density of 10^5^ cells/well, were transferred to 24-well tissue culture plates, in a total volume each of 500 μl. The cells were incubated in these conditions overnight, allowing the cells to achieve confluence. The following day, the 500 μl cell culture medium was removed and replaced with 300 μl of fresh medium containing test agent at the appropriate concentration. The cells were then returned to the incubator for 24 h.

EpiSkin™ 3D skin constructs were treated as recommended by the manufacturer (Fig. 1E). EpiSkin™ was exposed to the test compounds for 60 min. This exposure time was decided upon following communications with the manufacturer — 15 min exposure of EpiSkin™ was recommended for ‘raw materials’, with longer exposure times of 60 min recommended for ‘finished products’.

To assess cell viability, the cell monolayers were washed with 500 μl sterile PBS (pH 7.4) and were then replenished with MEM containing MTT (3-(4,5-dimethylthiazol-2-yl)-2,5-diphenyltetrazolium bromide) reagent at 1 mg ml^− 1^. The cells were then returned to the incubator for a final 2 h. The MTT assay is a gold-standard assay for determining cell viability and is based on the ability of viable cells to reduce the water-soluble MTT to a water-insoluble formazan product [22,23]. The cell supernatants, following 2 h incubation of the cells in the MTT containing medium, were discarded and 500 μl of dimethyl sulfoxide (DMSO) was added to each well of the 24 well plate to solubilise the formazan product. The plates were shaken for 2 min to assist solubilisation, before 200 μl aliquots were transferred to duplicate wells of a 96-well microplate for absorbance measurement. The absorbance was measured at 550 nm, with DMSO acting as a background control.

To assess the release of IL-1α into the cell culture medium in response to the various test stimuli, the MEM that was reserved in the viability test was used in a commercially available human IL-1α specific ELISA, following the manufacturer's guidelines. Additionally, cellular production (cell-associated IL-1α) of the same cytokine in the absence of cellular secretion was also investigated. The cells were similarly challenged and following treatment, were lysed in PBS (pH 7.4) containing 0.5% (v/v) Triton-X-100 on an upright carousel at 4 °C. Cell lysates were analysed for IL-1α content according to manufacturer's instructions [24]. The total protein content of the cell lysates was also assessed in order to normalise data in the case of cell-associated IL-1α measurement. This was performed using the standard Bradford assay (Pierce, Rockford, IL, USA) for protein concentration determination. IL-1α levels were expressed as pg IL-1α/μg total protein and were then converted to represent the relative IL-1α expression of the cells as a percentage of that expressed by those cells incubated under control conditions.

### 2.11 *In vivo* tolerance study

Tolerance studies using the PMVE/MA MN arrays were carried out prior to the commencement of *in vivo* experiments, in order to determine whether the formulation or the occlusive dressing to be used in subsequent experiments caused any irritation to the rats. Four MN arrays or pieces of occlusive dressings alone were applied using gentle finger pressure to the shaved backs of each of 2 rats and secured in place for 24 h with medical tape (Micropore^®^, 3 M, St. Paul, MN, USA). These were then removed following 24 h and the rats were monitored for any signs of irritation on their skin and any other adverse effects for up to 7 days post removal of the MN and/or occlusive dressing.

### 2.12 *In vivo* evaluation

All male *Sprague dawley* rats were acclimatised to laboratory conditions for a 7 day period. To prevent fur from interfering with dermal contact of the patch, animals were anesthetised using gas anaesthesia (2–4% isoflurane in oxygen) 24 h before experimentation, and the hair was removed with an animal hair clipper. Additionally, depilatory cream (Boots Expert®, The Boots Company PLC, Nottingham, UK) was used to remove any residual hair. This was followed by a one day recovery period to facilitate the re-establishment of skin barrier function [11,22]. The following day, the rats were again anaesthetised immediately prior to MN array application. Four dissolving MN arrays, which had each been secured onto an open “frame” of approximately 1.6 cm^2^ area made from adhesive foam (TG Eakin Ltd, Comber, Co. Down, UK) were applied opposite each other using firm finger pressure onto a pinched section of skin on the back of the rat, as illustrated in Fig. 5A. This orientation of application was used as a means to apply equal pressure to the two opposing arrays. An occlusive dressing was carefully applied over the MN arrays and these were then secured in place by gently wrapping the animal in Micropore^®^ tape, avoiding the legs. The rats were returned to their cages and tail vein bleeds (no more than 200 μl per sample) were collected into heparinised tubes at designated time points: 1, 2, 4, 6 and 24 h. The MN arrays were left in place for 24 h. Plasma was separated from the whole blood samples and prepared as described below, prior to HPLC analysis. Approval for animal experiments was obtained from the Committee of the Biological Research Unit, Queen's University Belfast. The work was carried out under the Project Licence PPL 2678 and the Personal Licence PIL 1466. All *in vivo* experiments were conducted according to the policy of the federation of European Laboratory Animal Science Associations and the European Convention for the protection of vertebrate animals used for experimental and other scientific purposes, with implementation of the principles of the 3R's (replacement, reduction, refinement).

### 2.13 Extraction of plasma and drug

Control rat blood for assay method development was obtained from healthy *Sprague dawley* rats. Blood from culled rats was collected *via* heart puncture with a heparinised syringe into ethylenediaminetetraacetic acid (EDTA)-coated tubes. Plasma separation was performed by centrifuging the blood at 500 ×*g* for 10 min in a refrigerated centrifuge (4 °C). The plasma was then aliquoted into microtubes and stored at − 80 °C until required. Aliquots (10 μl) of ibuprofen sodium working standard solutions were added to 190 μl blank plasma. The sample was vortex mixed for 10 s in a poly(propylene) microtube and 500 μl acetonitrile (ACN) was then added. The sample was vortex mixed for 10 min and centrifuged at 14,000 ×*g* for 10 min at 4 °C. The ACN extraction procedure was then repeated to ensure optimum extraction of the drug. The sample mixture was placed in a disposable glass culture tube and the extract was dried under a stream of nitrogen at 35 °C for 50 min using a Zymark TurboVap® LV Evaporator Workstation. The residue was then reconstituted in 200 μl PBS (pH 7.4) and collected into a microtube. This was then vortex mixed for 30 s and centrifuged at 14,000 ×*g* for 10 min at room temperature. The supernatant was transferred into an autosampler vial and 50 μl was injected onto the HPLC column.

### 2.14 Pharmaceutical analysis of ibuprofen sodium

Ibuprofen-sodium quantification in PBS and rat plasma was performed using reversed phase HPLC (Agilent 1200® Binary Pump, Agilent 1200®, Standard Autosampler, Agilent 1200® Variable Wavelength Detector, Agilent Technologies UK Ltd, Stockport, UK) with UV detection at 220 nm. Gradient separation was achieved using an Agilent Eclipse XDB-C18 (5 μm pore size, 4.6 × 150 mm) analytical column fitted with a guard cartridge of matching chemistry. The mobile phase was 60%:40% methanol:10 mM potassium phosphate (pH 4.6), with a flow rate of 1 ml min^− 1^ and a run time of 30 min per sample. The injection volume was 50 μl. The chromatograms obtained were analysed using Agilent ChemStation® Software B.02.01. Least squares linear regression analysis and correlation analysis were performed on the triplicate calibration curves produced on each of three separate days, enabling determination of the equation of the line, its coefficient of determination and the residual sum of squares (RSS). To determine the limit of detection (LoD) and limit of quantification (LoQ), an approach based on the standard deviation of the response and the slope of the representative calibration curve was employed, as described in the guidelines from the International Conference on Harmonisation (ICH) [19]. Ibuprofen sodium either dissolved in PBS (standards), or samples collected from the Franz cell apparatus (unknowns), was quantified by injection of the sample, following filter sterilisation through 0.2 μm filters, directly onto the HPLC column. In the case of plasma samples, the drug was first extracted from the plasma, as described above, and then the resulting sample, which had been reconstituted in PBS and filtered, was injected onto the column. The method parameters for detection of ibuprofen sodium in PBS and plasma were identical.

### 2.15 Statistical analysis

Where appropriate, data was analysed using the Student's *t*-test, one-way ANOVA with post-hoc comparisons, or Mann–Whitney *U*-test. In all cases, *p* < 0.05 denoted significance. Statistical Package for the Social Sciences, SPSS 18.0 version 2.0 (SPSS, Inc., Chicago, IL, USA), was used for all analyses.

## 3 Results

### 3.1 MN array fabrication

In this study, a range of commonly employed film-forming FDA-approved pharma polymers were investigated for their suitability in the preparation of a novel dissolving MN system loaded with high doses of ibuprofen-sodium. The MNs prepared have been summarised in Table 1, with brief accompanying comments relating to their identified properties. Some of the polymers tested in this study, used in various combinations and in various different drug loading ratios, were: Eudragit®L, Eudragit®S, poly(lactic acid) (PLA), poly(vinyl alcohol) (PVA), poly(vinylpyrrolidone) (PVP), Gantrez® MS-955 and alginic acid. For a variety of reasons, outlined in Table 1, the vast majority of the polymers investigated were not suitable for use in production of the intended drug-containing MN arrays. The co-polymer which showed the most promise and potential was the copolymer, poly(methyl vinyl ether/maleic acid) (PMVE/MA), prepared by aqueous hydrolysis of the parent anhydride Gantrez® AN-139.

Drug-loaded MN arrays were then formulated using a range of PMVE/MA stock concentrations at either pH 2.0 or pH 7.0, in combination with a range of different ibuprofen sodium loadings, ranging between 5 and 50% w/w. The formulation that produced the MN arrays with the most desirable characteristics, in terms of mechanical properties, as well as the highest achievable drug loading was fabricated in a ratio of 70% PMVE/MA 30% w/w gel, pH 7.0:30% ibuprofen sodium. Fig. 1B is a representative digital photograph of a dissolving MN array in a 19 × 19 format, therefore totalling 361 individual MN projections per array, each with height of 600 μm, base width of 300 μm and interspacing of 50 μm on an array area of approximately 0.49 cm^2^. These MN arrays were prepared using approximately 300 mg of the gel. Upon drying, approximately 40% of this mass was lost due to evaporation of the water contained within, resulting in arrays containing approximately 50% w/w active agent.

### 3.2 Rheological characterisation

**Fig. 2:**
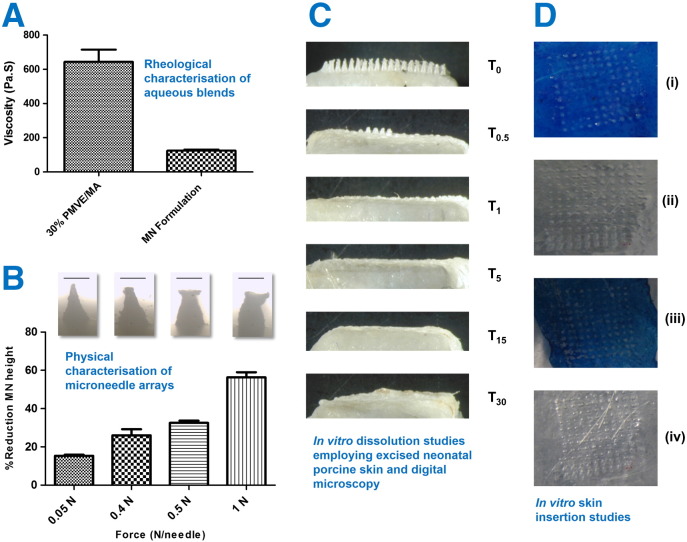
Viscosity of 30% w/w PMVE/MA, pH 7.0 gel formulation and the 70% PMVE/MA 30% w/w gel, pH 7.0:30% ibuprofen sodium formulation (optimised microneedle formulation), (means ± S.D., n = 5) (A). Digital microscope images of dissolving PMVE/MA microneedles loaded with ibuprofen sodium following the application of different forces (0.05, 0.4, 0.5 and 1.0 N/needle). Microneedle arrays were attached to the moveable cylindrical probe (length 5.0 cm, cross-sectional area 1.5 cm^2^) of the Texture Analyser using double-sided adhesive tape and an axial compression load was then applied. The test station pressed the MN arrays against a flat block of aluminium at a rate of 0.5 mm s^− 1^ with defined forces for 30 s. The pre- and post-test speeds were 1.0 mm s^− 1^ and the trigger force was set at 0.049 N. These images are representative of the percentage reduction in the heights of needles on the MN arrays observed following the application of the different forces (means + S.D., n = 3). The scale bars represent a length of 300 μm (B). Representative digital micrographs illustrative of the dissolution of ibuprofen sodium-loaded dissolving microneedle arrays (prepared from gels comprised of 70% PMVE/MA 30% w/w gel, pH 7.0:30% ibuprofen sodium) at specific time points (T_0_: 0 min; T_0.5_: 0.5 min; T_1_: 1 min *etc.*) over a 30 min period following insertion into, and removal from excised neonatal porcine skin (C). Digital images showing microconduits on methylene-blue stained neonatal porcine skin after application of forces of (i) 0.4 & (iii) 0.5 N/needle to ibuprofen sodium-loaded dissolving PMVE/MA microneedle arrays. The images presented in (ii) and (iv) are the reciprocal images of the etching visible on the laboratory film (Parafilm®) lying under the microneedle arrays following force application (D).

The rheological and mechanical properties of the gel formed when the candidate polymer, PMVE/MA was mixed with ibuprofen sodium (70% PMVE/MA 30% w/w, pH 7.0:30% ibuprofen sodium) were deduced. The viscosity of a polymer solution depends on concentration, size (*i.e.* molecular weight) of the dissolved polymer, pH, temperature, ionic strength and additives at a given shear rate. The viscosity of 30% w/w PMVE/MA, pH 7.0 decreased significantly (*p* < 0.001) after the addition of the ibuprofen sodium. This was possibly due to dampening of electrostatic repulsion between the ionisable free acid groups caused by the presence of the additional sodium content of the gels formed (Fig. 2A).

### 3.3 Mechanical testing of MN arrays

The Texture Analyser was used to determine the mechanical strength of the ibuprofen sodium-containing PMVE/MA MN arrays. As applied force was increased, there was a progressive decrease in MN height but, notably, none of the MN fractured, rather they became somewhat compressed (Fig. 2B). In general, increasing the forces applied per needle resulted in an increase in the penetration efficiency of the arrays, as shown in Fig. 2D. The percentage of microconduits successfully created in the skin at insertion forces of 0.40 and 0.50 N/needle were approximately 90% and 100%, respectively. In both cases, microconduits could also be traced on the surface of the laboratory film (Parafilm®) placed underneath the dermatomed neonatal porcine skin, Fig. 2D (ii) and (iv).

### 3.4 Water content determination and ibuprofen sodium content

**Fig. 3:**
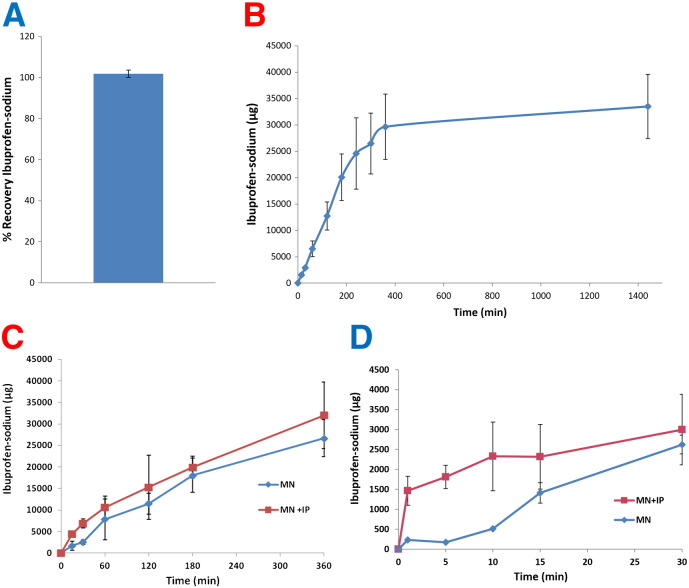
Percentage recovery of ibuprofen sodium from dissolving PMVE/MA microneedle arrays (means ± S.D., n = 6) (A). The *in vitro* cumulative permeation profile of ibuprofen sodium across dermatomed 350 μm neonatal porcine skin when delivered using in-dwelling dissolving PMVE/MA microneedle arrays (means ± S.D., n = 6) (B). The inset chart shows the mass balance following completion of the 24 h delivery experiment as compared to the theoretical loading of ibuprofen sodium. The *in vitro* cumulative permeation profile of ibuprofen-sodium across dermatomed 350 μm neonatal porcine skin when combining iontophoresis (0.5 mA cm^− 2^) and in-dwelling dissolving PMVE/MA microneedle arrays for a period of 6 h (MN + IP), or passive delivery from dissolving microneedle arrays only (MN) (means ± S.D., n = 5) (C). The *in vitro* cumulative permeation profile of ibuprofen-sodium across dermatomed 350 μm neonatal porcine skin when combining iontophoresis (0.5 mA cm^− 2^) and in-dwelling dissolving PMVE/MA microneedle arrays for a period of 30 min (MN + IP), or passive delivery from dissolving microneedle arrays only (MN) (means ± S.D., n = 5) (D).

The bound and free water of the ibuprofen sodium-containing PMVE/MA MN arrays, as determined using thermogravimetric analysis, were 7.22 ± 0.64% and 3.50 ± 0.53%, respectively (means ± S.D., n = 3). The percentage recovery of ibuprofen sodium from MN arrays which were dissolved post-casting in a known volume of PBS (pH 7.4) was 99.6 ± 0.46% (means ± S.D., n = 6) (Fig. 3A).

### 3.5 In-skin dissolution studies

Dissolution of the needles was evident after as little as 30 s and the needles had completely disappeared after 5 min. An additional dissolution study was carried out in which dissolving MN arrays were placed into flasks containing 20 ml PBS (pH 7.4). The dissolution time for MN arrays was 4.39 ± 0.88 min. These experiments indicate the rapidity of MN dissolution using this drug delivery system.

### 3.6 Pharmaceutical analysis of ibuprofen sodium

HPLC methods for the quantification of ibuprofen-sodium, in either PBS or plasma, were validated according to ICH guidelines. Limits of quantification and detection were determined and are presented in Table 2(i) and (ii), respectively.

### 3.7 *In vitro* drug delivery from ibuprofen sodium-loaded dissolving PMVE/MA MN arrays

Ibuprofen sodium was detected in the receiver compartment of the Franz cells by HPLC analysis 15 min following application of MN arrays and the concentration of drug in the cells increased in a time-dependent manner until the end of the release experiment (24 h) (Fig. 3B). MN arrays delivered a total of 33.5 ± 6 mg of ibuprofen sodium in 24 h. The MN arrays contained an average total of 37.24 ± 2.25 mg ibuprofen sodium, meaning that approximately 90% of the contained drug loading was delivered across the dermatomed neonatal porcine skin in 24 h.

### 3.8 Cathodal iontophoresis

Cathodal iontophoresis coupled with the utilisation of the dissolving MN delivery system may result in increased rates of skin permeation. This was investigated here and Fig. 3C illustrates the permeation profiles of ibuprofen sodium following cathodal iontophoresis (IP), at a current density and duration of 0.5 mA cm^− 2^ and 6 h, respectively. The combination of cathodal IP with this dissolving MN delivery system led to an enhancement in ibuprofen sodium permeation across the dermatomed neonatal porcine skin over a 6 h sampling period, in comparison to the dissolving MN array used in isolation. This difference was not significant, however (*p* > 0.05) as 26.65 ± 0.43 mg of drug was delivered in the case of MN arrays used in isolation, as compared to 31.98 ± 0.78 mg of drug delivered when MN arrays and IP were used in combination. The *in vitro* permeation of ibuprofen sodium using the combinatorial approach of dissolving MN arrays and cathodal IP was then followed for a shorter period of 30 min only (Fig. 3D). The quantity of ibuprofen sodium which was successfully delivered using the combination of dissolving MN arrays and cathodal IP was significantly higher (*p* < 0.05) than that achieved with MN arrays only at 1, 5 and 10 min. Following these initial sampling time points, there was no significant difference between the quantities of drug delivered at subsequent time points using the two different delivery strategies. Accordingly, due to its limited value and impractical nature, IP was discontinued at this stage.

### 3.9 Biocompatibility studies

**Fig. 4:**
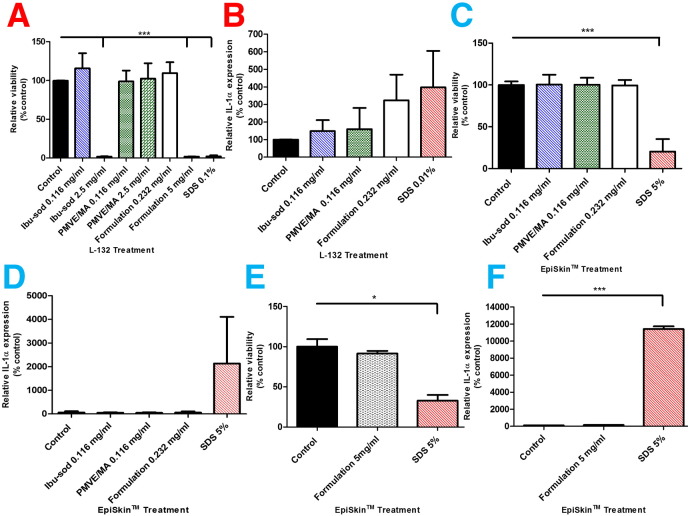
Effects of 24 h exposure, on the viability of human L-132 fibroblasts, to the indicated treatments: Cells incubated in cell culture medium only (Control); ibuprofen-sodium only (Ibu-sod); PMVE/MA only (PMVE/MA); MN array formulation (formulation) or SDS positive control (SDS). Cell viability following exposure was expressed relative to the viability of cells in the control group (means ± S.D., n = 3) (***p = 0.0001) (A). IL-1α content of L-132 cell lysates following 24 h exposure to indicated treatments: Cells incubated in cell culture medium only (control); ibuprofen-sodium only (Ibu-sod); PMVE/MA only (PMVE/MA); MN array formulation (formulation) or SDS positive control (SDS) (means ± S.D., n = 3) (B). The viability of human keratinocytes in 3D culture (EpiSkin™) (n = 4) following treatment for 60 min, followed by 42 h recovery with the indicated treatments: Cells incubated in cell culture medium only (control); ibuprofen-sodium only (Ibu-sod); PMVE/MA only (PMVE/MA); MN array formulation (formulation) or SDS positive control (SDS). (means ± S.D., n = 4) (***p = 0.0001) (C). IL-1α content of human keratinocytes in 3D culture (EpiSkin™) following treatment for 60 min, followed by 42 h recovery with the indicated treatments: Cells incubated in cell culture medium with a relevant volume of PBS (control); ibuprofen-sodium only (Ibu-sod); PMVE/MA only (PMVE/MA); MN array formulation (formulation) or SDS positive control (SDS), (means ± S.D., n = 4) (D). The viability of human keratinocytes in 3D culture (EpiSkin™) following treatment for 60 min, followed by 42 h recovery with the indicated treatments: MN array formulation (formulation), SDS positive control (SDS) or cells incubated in cell culture medium with a relevant volume of PBS pH 7.4 (negative control), (means ± S.D., n = 4) (*p = 0.0153) (E). IL-1α content of human keratinocytes in 3D culture (EpiSkin™) following treatment for 60 min, followed by 42 h recovery with the indicated treatments: Cells incubated in cell culture medium with a relevant volume of PBS (control); MN array formulation (formulation); SDS positive control (SDS) (means ± S.D., n = 4) (***p = 0.0004) (F).

Two concentrations of each of PMVE/MA and ibuprofen sodium were used in all biocompatibility tests, namely 0.116 and 2.5 mg ml^− 1^, while two concentrations of dissolved MN formulation were also employed; namely 0.232 and 5 mg ml^− 1^. In *in vitro* delivery experiments, the concentration of ibuprofen sodium in the receiver compartment after 15 min was approximately 0.116 mg ml^− 1^ and that after 6 h was approximately 2.5 mg ml^− 1^. These concentrations were doubled for the dissolved MN formulation to reflect the high drug and polymer loadings (approximately 90% in total) and the fact that the mechanism of action of the device involves deposition of both in the skin. Exposure of L-132 lung fibroblasts, used an epithelial cell model in this study, to low concentrations of ibuprofen sodium (0.116 mg ml^− 1^), PMVE/MA (0.116 mg ml^− 1^) or MN formulation (0.232 mg ml^− 1^) had no detrimental effects on cell viability (Fig. 4A). Exposure to the higher concentration of PMVE/MA (2.5 mg ml^− 1^) also did not lead to a significant reduction in cell viability. Exposure to high concentrations of ibuprofen sodium (2.5 mg ml^− 1^) and the MN formulation (5 mg ml^− 1^) negatively impacted cell viabilities, as the relative viability of the cells decreased to 1.8 ± 0.4% and 1.8 ± 0.2%, respectively (Fig. 4A). These decreases were deemed to be significant (*p* < 0.0001). A positive control of 0.1% w/v SDS unsurprisingly also led to a significant (*p* < 0.0001) reduction in cell viability.

Treatment of L-132 fibroblasts for 24 h with a 0.01% w/v solution of SDS in MEM resulted in a four-fold increase in the amount of cell-associated IL-1α (Fig. 4B). Exposure of the cells to 0.116 mg ml^− 1^ ibuprofen sodium or PMVE/MA led to marginal, but not significant, increases in IL-1α expression. Increasing the concentration of the MN formulation to 0.232 mg ml^− 1^ led to an increase in the expression of the inflammatory marker, but this was not significant (*p* > 0.05). Treatment with 2.5 mg ml^− 1^ ibuprofen sodium and 5 mg ml^− 1^ MN formulation had resulted in almost total loss of L-132 viability (Fig. 4A). Accordingly, these solutions led to undetectable cell-associated IL-1α levels in the cell lysates (Fig. 4B). No IL-1α was detectable in the culture medium itself following treatment, indicating that the IL-1α was not secreted by the cells. The data indicates that the drug and co-polymer, used in isolation or in combination at the indicated concentrations, were not irritant to the cells. Treatment of EpiSkin, reconstructed human epidermis, with 5% w/v SDS (the recommended positive control) for 60 min followed by a 42 h recovery period, resulted in an 80% loss of tissue viability (*p* < 0.0001) (Fig. 4C). Similar treatments with ibuprofen sodium, PMVE/MA (both 0.116 mg ml^− 1^) or MN formulation (0.232 mg ml^− 1^) had no deleterious effects on tissue viability (Fig. 4C). Analysis of the IL-1α content of the medium that contained the tissue revealed that treatment with 5% w/v SDS caused a 2000% increase in cytokine production relative to the negative control (Fig. 4D). The other treatments did not affect expression of the cytokine. All exposures were carried out for 60 min, followed by a 42 h recovery period, according to the manufacturers' recommendations. Tissues were then exposed to the higher concentration of the MN formulation, namely 5 mg ml^− 1^, for 60 min followed by a 42 h recovery period. This treatment of the tissues did not have any deleterious effects on their viability, while treatment with 5% w/v SDS caused a significant decrease in cell viability (*p* = 0.0153) (Fig. 4E). In the reciprocal IL-1α expression experiment, the IL-1α content of the medium was unaffected by treatment with 5 mg ml^− 1^ of the MN formulation. In stark contrast, however, there was a marked increase in IL-1α expression (11,411 ± 316% relative to the negative control, *p* = 0.0004) by those cells treated with the positive control of 5% w/v SDS (Fig. 4F). The results of the work carried out using both 2D and 3D cultures outlined here was further underpinned by the results of the tolerance studies carried out in rats prior to the commencement of *in vivo* delivery of ibuprofen sodium, whereby the application of the dissolving MN arrays held in place using occlusive dressings for 24 h caused no irritation to the animals. The results of these two tolerance studies indicated that *in vivo* experiments could, therefore, commence.

### 3.10 *In vivo* drug delivery and quantification

**Fig. 5:**
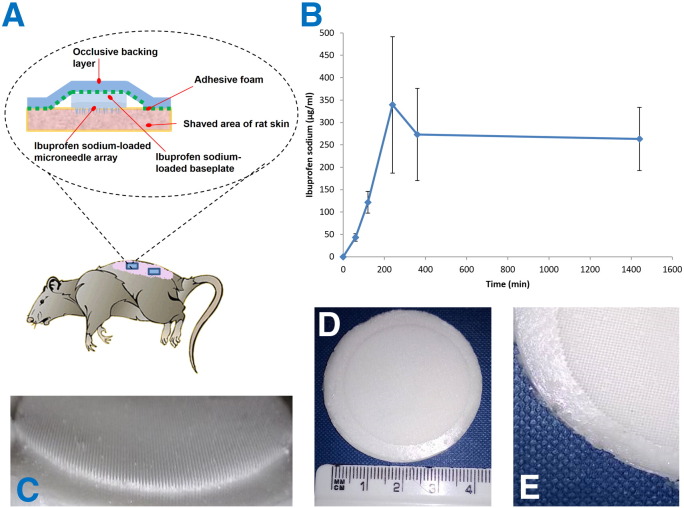
Schematic representation of application and retention strategies for rat experiments designed to evaluate *in vivo* performance of dissolving PMVE/MA microneedle arrays (A). The *in vivo* plasma profiles of ibuprofen sodium (means ± S.D., n = 4) following transdermal delivery using dissolving PMVE/MA microneedle arrays (B). Images of a 10 cm^2^ ibuprofen sodium-loaded PMVE/MA dissolving microneedle patch and two closer images of the same array where the individual needles on the array are clearly visible (C–E).

The most appropriate site for the application of the dissolving MN arrays was determined to be the back, adjacent to the spinal region, an area less likely to be easily scratched/groomed by the animal. In addition, this area was chosen so as to ensure that flexing of the abdominal cavity due to normal breathing and movement could not potentially cause MN arrays to detach from the skin during the course of experiments. The application and positioning of the MN arrays on the backs of the animals is exemplified in Fig. 5A.

It was quickly deduced that MN arrays applied without the adhesive foam frame had a tendency to become expelled out of the skin upon release of the application pressure and loosening of the pinched skin. Therefore, in all *in vivo* experimentation, these adhesive frames were employed to secure the arrays in place over the course of the experiment. Following application of the dissolving MN arrays, a transdermal occlusive dressing was applied, covering and surrounding the arrays so as to prevent the animals from removing them during routine grooming. Four dissolving MN arrays were applied to the backs of each of four rats in any given experiment. The average ibuprofen sodium content in each dissolving MN array was approximately 76.4 mg. Therefore, as each rat had four dissolving MN arrays applied, each had an average total of 305.6 mg ibuprofen sodium applied in the experiment. As is clearly shown in Fig. 5B, the use of dissolving MN arrays to transdermally administer ibuprofen sodium resulted in a progressive increase in the plasma concentrations of the drug within the initial 4 h sampling period, with a maximal concentration of approximately 339 μg ml^− 1^ achieved within that timeframe. Furthermore, the use of these dissolving MN arrays enabled sustained transdermal delivery of ibuprofen sodium over the 24 h investigation period, resulting in a final plasma concentration of approximately 263 μg ml^− 1^.

## 4 Discussion

The data presented here represents the first time a high dose low molecular weight drug molecule has been delivered using dissolving microneedles to yield therapeutically-relevant plasma levels. The transdermal route has already been extensively investigated as a potential means of non-invasive administration of a range of non-steroidal anti-inflammatory drugs (NSAIDs), all of which have relatively high daily doses. The major challenge associated with transdermal NSAID delivery to date has been poor permeation across the *stratum corneum*. Dissolving MN arrays, capable of breaching the skin's outermost layer to enhance drug delivery, was the focus of the present study. A wide range of different polymeric formulations were cast into MN moulds and provisionally assessed for mechanical soundness and polymer/drug homogeneity. The copolymer which showed the most potential was PMVE/MA. Blends of ibuprofen sodium/PMVE/MA were prepared and used to fabricate MN arrays. In contrast to all other formulations investigated here, the mechanical properties and homogeneity of the resultant arrays prepared from gels comprising 70% w/w of a 30% w/w PMVE/MA gel neutralised to pH 7.0 and 30% w/w ibuprofen-sodium were satisfactory. This formulation was, thus, chosen for subsequent study. These MN arrays successfully penetrated excised neonatal porcine skin and had a sufficiently high drug content to yield suitable *in vitro* and *in vivo* delivery. Increasing the drug content above 30% w/w produced arrays with a more powder-like consistency and the MN arrays were incapable of penetrating the skin. Formulations incorporating lower drug content were sufficiently rigid to penetrate the skin, but lacked the high NSAID content essential to achieving the drug delivery target. The dissolving MN arrays prepared in this study were capable of penetrating the *SC* of the skin at relatively low forces. Following insertion into the skin, it is of fundamental importance that the behaviour of the dissolving MN systems is characterised and understood. Previously reported studies have shown that the complete dissolution of soluble MN arrays can range from minutes to hours, depending upon the composition and water solubility of the polymeric system employed [4,25–27]. The delivery of ibuprofen sodium to the skin is based upon the dissolution of the polymeric MN arrays following exposure to the interstitial fluid. The dissolution of the individual needles on an array upon insertion into neonatal porcine skin in this study took less than 5 min. Although topical ibuprofen preparations are marketed, evidence has shown that these gels are unsuitable for systemic delivery of the anti-inflammatory [28]. One report suggested that rival ibuprofen-containing topical products varied five-fold in terms of ibuprofen delivery capabilities [29]. The high drug content of the MN formulation investigated in the present study represents a clear advantage of this polymeric MN delivery system over the typically low drug content of topical applications (5–10% w/w). Previous studies have demonstrated the synergistic effect of MN pre-treatment of skin in conjunction with topical application of NSAIDs in enhancing transdermal delivery of other drugs [30], but the current study is the first to achieve the delivery of therapeutically-relevant concentrations of ibuprofen to plasma. The *in vivo* rat studies have yielded results that could ultimately prove important in progressing transdermal drug delivery. Delivery of ibuprofen sodium from dissolving MN arrays yielded extremely high and sustained blood plasma concentrations. The therapeutic blood levels of ibuprofen in humans range between 10 and 15 μg/ml [31]. Based on this knowledge and our *in vivo* results, we can cautiously approximate the patch size that might be necessary for use in human volunteers, appreciating the known differences in rat and human skin. An average human male weighs approximately 60 kg [32], which is 180 times greater than the weight of a 340 g rat (the average weight of rats used in these experiments). The blood plasma ibuprofen-sodium concentration achieved in the rats (263 μg ml^− 1^ at the 24 h time point) were approximately 20 times greater than the human therapeutic plasma level and this was achieved with MN arrays of total approximate area of approximately 2 cm^2^ (4 × 0.49 cm^2^). By this rationale, a MN patch design of no greater than 10 cm^2^ could potentially deliver therapeutically relevant concentrations of ibuprofen sodium in humans. Typical commercialised transdermal patches can be as large as 30 or 40 cm^2^ (Novartis make Nicotinell® nicotine patches of 30 cm^2^ [33]; Janssen make Duragesic® CII (fentanyl) patches of 32 and 42 cm^2^ [34]). Accordingly, it is reasonable to suggest that, following further evaluation, first in pigs and then human volunteers, an optimised MN product could ultimately be developed based on the technology presented here. Indeed, Fig. 5(C–E) shows a 10 cm^2^ patch made from our ibuprofen sodium-loaded PMVE formulation. Although treatment of L-132 cell monolayers with 2.5 mg ml^− 1^ ibuprofen-sodium led to a decrease in cell viability, it is extremely unlikely that the viable skin cells would be exposed to such high concentrations of ibuprofen sodium *in vivo* following MN application, as the fast dissolution kinetics of the MN arrays, coupled with the ability of the rich dermal microcirculation to rapidly absorb drug substances from the skin [14,22], would expedite the removal of the active from the local area. This observation is also of interpretational relevance in the case of L-132 cells treated with 0.232 mg ml^− 1^ MN formulation, which resulted in increased release of the irritancy biomarker, while absolutely no skin irritation was observed in rats. Treatment of human keratinocytes in 3D culture (EpiSkin™) with the dissolving MN formulation (up to 5 mg ml^− 1^) caused no significant reduction in the viability of these cells and no skin irritancy was caused by use of the MN system in the *in vivo* model, thus corroborating these points. It is notable that EpiSkin™ was endorsed by the European Centre for the Validation of Alternative Methods (ECVAM)'s Scientific Advisory Committee (ESAC) as ‘*a reliable and relevant stand*-*alone test for predicting rabbit skin irritation*’, and is seen as a valid replacement for animal studies of skin irritancy. EpiSkin™ was the only *in vitro* model so endorsed. The EpiSkin™ model is suggested as a suitable candidate for *in vitro* screening for skin irritation in ISO 10993-10 (Biological evaluation of medical devices — Part 10: Tests for irritation and skin sensitization). A loss of 50% viability following treatment and recovery is deemed to constitute irritancy according to the Organisation for Economic Co-operation and Development (OECD) Test Guideline 439 (*In Vitro* Skin Irritation: Reconstructed Human Epidermis Test Method). To this effect, 5% w/v SDS treatment of EpiSkin cultures resulted in a 62% drop in cell viability, identifying it as an irritant. The MN formulation reported here (5 mg ml^− 1^), therefore, lies comfortably within the non-irritant range, with a loss of only 8.4% cell viability following treatment. In previous work carried out by our Group, the large-molecule payload of the dissolving MN arrays which was successfully delivered *in vitro* was confined to that amount of the active (insulin) contained within the individual needles of the arrays themselves [11]. In the current study however, this was not the case. The delivery of 1530 μg ibuprofen sodium, the theoretical mass of ibuprofen sodium contained within the dry MNs alone, was vastly exceeded over the course of the experimental period. In the *in vitro* studies, approximately 33 mg of the drug initially loaded into the arrays was delivered. This equates to approximately 90% of the total loaded drug being successfully delivered over the 24 h experimental period and demonstrates the significant potential of dissolving MN arrays in facilitated transdermal delivery of high dose low molecular weight drugs.

## 5 Conclusion

The work presented here illustrates the potential of appropriately-formulated dissolving microneedles to deliver a low molecular weight, high dose drug in therapeutically-relevant doses. This technology could potentially be harnessed to deliver large quantities of such non-potent therapeutic agents across the skin's barrier layer without causing irritation. In this way, a common limitation of transdermal delivery, namely low delivery capacity for non-potent drugs, could possibly be overcome. This work, therefore, represents a significant progression in the utilisation of microneedle technologies for successful transdermal delivery of a much wider range of drugs. We are currently progressing towards clinical evaluations with a range of candidate molecules and are taking regulatory advice, given that at least 5 mg of polymer is deposited in each square centimetre of skin with this system. Investigation of accumulation/distribution and elimination of polymer from such a dissolving microneedle product designed for regular use is likely to be essential before regulatory approval.

## Figures and Tables

**Table 1:** Summary of the content of the various aqueous blends tested in the production of dissolving MN arrays loaded with ibuprofen sodium. In all cases, representative images of the formed MN are provided.

**50% w/w PLA:50% w/w ibuprofen-sodium**			**21% w/w Eudragit®L:30% w/w ibuprofen-sodium**
Stamp moulded MN arrays tacky			Needles on the MN arrays were shallow and did not form completely.
**18% w/w Eudragit®S:30% w/w ibuprofen-sodium**			**21% w/w PVA:30% w/w ibuprofen-sodium**
There was distinct separation of drug and polymer in the MN arrays			Insoluble drug/polymer aggregates formed in the polymer gel and subsequently in the MN arrays.
**21% w/w PVA:30% w/w ibuprofen-sodium**			**14% w/w Gantrez®MS-955, pH 6.0:30% w/w ibuprofen-sodium**
Insoluble drug/polymer aggregates formed in the polymer gel and subsequently in the MN arrays.			MN arrays were extremely brittle and needles did not form entirely.
**70% w/w alginic acid:30% w/w ibuprofen-sodium**			**10.5% w/w alginic acid:30% w/w ibuprofen-sodium**
The polymer/drug mix was not homogenous and MN arrays were subsequently brittle.			Needles broke off the MN arrays as they were removed from moulds.
**3% alginic acid:40% w/wibuprofen-sodium**			**18% w/w PVP, pH 6.0:40% w/w ibuprofen-sodium**
MN arrays were extremely brittle when removed from moulds.			MN arrays were extremely brittle.
**18% w/w PVP:1.5% w/w Eudragit®L, pH 7.0:40% w/w ibuprofen-sodium**			**15% w/w PVP:6% w/w Eudragit®S pH 7.0:40% w/w ibuprofen-sodium**
Needles did not form and MN arrays were extremely brittle.			MN arrays were brittle and needles did not form.
**18% w/w PVP:0.75% w/w Eudragit®S:40% w/w ibuprofen-sodium**			**18% w/w PVP:0.75% w/w Eudragit®L:40% w/w ibuprofen-sodium**
MN arrays were extremely brittle and broke upon removal from the moulds.			Needles were fragile and broke off when MN arrays were removed from moulds.
**18% w/w PVP:1.5% w/w Eudragit®L:40% w/w ibuprofen-sodium**			**15% w/w PVP:6% w/w Eudragit®S:40% w/w ibuprofen-sodium**
Needles were fragile and broke off when MN arrays were removed from moulds.			Drug was insoluble in the polymer gel.
**12% w/w PVA:40% w/w ibuprofen-sodium**			
Needles were fragile and broke off when MN arrays were removed from moulds.			

**Table 2:** Calibration curve properties for ibuprofen sodium quantification in (i) PBS (pH 7.4) and (ii) rat plasma, as determined by linear regression and correlation analyses, and limits of detection and quantification for ibuprofen sodium.

	Slope	y-Intercept	R^2^	LoD (μg/ml)	LoQ (μg/ml)
(i)	103.27	10.576	0.9996	0.992	1.25
(ii)	109.83	17.876	0.9991	0.7	2.2

## References

[1] Ito Y., Hirono M., Fukushima K., Sugioka N., Takada K. (2012). Two-layered dissolving microneedles formulated with intermediate-acting insulin. Int. J. Pharm..

[2] Liu S., Jin M.N., Quan Y.S., Kamiyama F., Katsumi H., Sakane T., Yamamoto A. (2012). The development and characteristics of novel microneedle arrays fabricated from hyaluronic acid, and their application in the transdermal delivery of insulin. J. Control. Release.

[3] Donnelly R.F., Morrow D.I.J., Singh T.R.R., Migalska K., McCarron P.A., O'Mahony C., Woolfson A.D. (2009). Processing difficulties and instability of carbohydrate microneedle arrays. Drug Dev. Ind. Pharm..

[4] Lee J.W., Park J.H., Prausnitz M.R. (2008). Dissolving microneedles for transdermal drug delivery. Biomaterials.

[5] Gomaa Y.A., Garland M.J., McInnes F., El-Khordagui L.K., Wilson C., Donnelly R.F. (2012). Laser-engineered dissolving microneedles for active transdermal delivery of nadroparin calcium. Eur. J. Pharm. Biopharm..

[6] Matsuo K., Yokota Y., Zhai Y., Quan Y.S., Kamiyama F., Mukai Y., Okada N., Nakagawa S. (2012). A low-invasive and effective transcutaneous immunization system using a novel dissolving microneedle array for soluble and particulate antigens. J. Control. Release.

[7] Naito S., Ito Y., Kiyohara T., Kataoka M., Ochiai M., Takada K. (2012). Antigen-loaded dissolving microneedle array as a novel tool for percutaneous vaccination. Vaccine.

[8] Sullivan S.P., Koutsonanos D.G., Del Pilar Martin M., Lee J.W., Zarnitsyn V., Choi S.O., Murth N., Compans R.W., Skountzou I., Prausnitz M.R. (2010). Dissolving polymer microneedle patches for influenza vaccination. Nat. Med..

[9] Matsuo K., Hirobe S., Yokota Y., Ayabe Y., Seto M., Quan Y.S., Kamiyama F., Tougan T., Horii T., Mukai Y., Okada N., Nakagawa S. (2012). Transcutaneous immunization using a dissolving microneedle array protects against tetanus, diphtheria, malaria, and influenza. J. Control. Release.

[10] Garland M.J., Caffarel-Salvador E., Migalska K., Woolfson A.D., Donnelly R.F. (2012). Dissolving polymeric microneedle arrays for electrically assisted transdermal drug delivery. J. Control. Release.

[11] Migalska K., Morrow D.I., Garland M.J., Thakur R., Woolfson A.D., Donnelly R.F. (2011). Laser-engineered dissolving microneedle arrays for transdermal macromolecular drug delivery. Pharm. Res..

[12] McCrudden M.T., Singh T.R., Migalska K., Donnelly R.F. (2013). Strategies for enhanced peptide and protein delivery. Ther. Deliv..

[13] Tuan-Mahmood T.M., McCrudden M.T.C., Torrisi B.M., McAlister E., Garland M.J., Singh T.R., Donnelly R.F. (2013). Microneedles for intradermal and transdermal drug delivery. Eur. J. Pharm. Sci..

[14] Benson H.A.E., Watkinson A.C. (2012). Topical and Transdermal Drug Delivery: Principles and Practice.

[15] Donnelly R.F., Majithiya R., Singh T.R., Morrow D.I., Garland M.J., Demir Y.K., Migalska K., Ryan E., Gillen D., Scott C.J., Woolfson A.D. (2011). Design, optimization and characterisation of polymeric microneedle arrays prepared by a novel laser-based micromoulding technique. Pharm. Res..

[16] Donnelly R.F., Morrow D.I., Fay F., Scott C.J., Abdelghany S., Singh R.R., Garland M.J., Woolfson A.D. (2010). Microneedle-mediated intradermal nanoparticle delivery: potential for enhanced local administration of hydrophobic pre-formed photosensitisers. Photodiagn. Photodyn. Ther..

[17] Woolfson A.D., McCafferty D.F., McCallion C.R., McAdams E.T., Mc J., Anderson C. (1995). Moisture activated, electrically conducting bioadhesive hydrogels as interfaces for bioelectrodes: effect of film hydration on cutaneous adherence in wet environments. J. Appl. Polym. Sci..

[18] Fourtanier A., Berrebi C. (1989). Miniature pig as an animal model to study photoaging. Photochem. Photobiol..

[19] (2005). International conference on harmonisation of technical requirements for registration of pharmaceuticals for human use. ICH Harmonised Tripartite Guideline — Validation of Analytical Procedures: Text and Methodology — Q2(R1).

[20] Dickson F.M., Lawrence J.N., Benford D.J. (1993). Surfactant-induced cytotoxicity in cultures of human keratinocytes and a commercially available cell line (3T3). Toxicol. *In Vitro*.

[21] Martinez V., Corsini E., Mitjans M., Pinazo A., Vinardell M.P. (2006). Evaluation of eye and skin irritation of arginine-derivative surfactants using different *in vitro* endpoints as alternatives to the *in vivo* assays. Toxicol. Lett..

[22] Donnelly R.F., Singh T.R., Garland M.J., Migalska K., Majithiya R., McCrudden C.M., Kole P.L., Mahmood T.M., McCarthy H.O., Woolfson A.D. (2012). Hydrogel-forming microneedle arrays for enhanced transdermal drug delivery. Adv. Funct. Mater..

[23] Mosmann T. (1983). Rapid colorimetric assay for cellular growth and survival: application to proliferation and cytotoxicity assays. J. Immunol. Methods.

[24] Ahn J.H., Eum K.H., Lee M. (2010). Assessment of the dermal and ocular irritation potential of lomefloxacin by using in vitro methods. Toxicol. Res..

[25] Lee J.W., Choi S.O., Felner E.I., Prausnitz M.R. (2011). Dissolving microneedle patch for transdermal delivery of human growth hormone. Small.

[26] Chu L.Y., Choi S., Prausnitz M.R. (2010). Fabrication of dissolving polymer microneedles for controlled drug encapsulation and delivery: bubble and pedestal microneedle designs. J. Pharm. Sci..

[27] Lee K., Lee C.Y., Jung H. (2011). Dissolving microneedles for transdermal drug administration prepared by stepwise controlled drawing of maltose. Biomaterials.

[28] Jorge L.L., Feres C.C., Teles V.E. (2010). Topical preparations for pain relief: efficacy and patient adherence. J Pain Res..

[29] Hadgraft J., Whitefield M., Rosher P.H. (2003). Skin penetration of topical formulations of ibuprofen 5%: an *in vitro* comparative study. Skin Pharmacol. Appl. Skin Physiol..

[30] Stahl J., Wohlert M., Kietzmann M. (2012). Microneedle pretreatment enhances the percutaneous permeation of hydrophilic compounds with high melting points. BMC Pharmacol. Toxicol..

[31] Dollery C. (1999). Therapeutic drugs.

[32] NHS website http://www.nhs.uk/Livewell/healthy-living/Pages/height-weight-chart.aspx.

[33] Nicotinell website http://www.nicotinell.co.uk/.

[34] Duragesic website http://www.duragesic.com/.

